# Neurovascular Coupling, Gait Parameters, and Cognitive Performance as Biomarkers of Mild Cognitive Impairment

**DOI:** 10.1093/geroni/igaf122.4263

**Published:** 2025-12-31

**Authors:** Yihui Cai, Wayne Chan, Tongyu Ma, Kaixin Zhou, Yarou Yuan, Shuning Li, Marco Pang

**Affiliations:** The Hong Kong Polytechnic University, Hong Kong, China (People’s Republic); The Hong Kong Polytechnic University, Hong Kong, China (People’s Republic); The Hong Kong Polytechnic University, Hong Kong, China (People’s Republic); The Second Clinical School of Clinical Medicine of Guangzhou, University of Chinese Medicine, Guangzhou, Guangdong, China (People’s Republic); The Hong Kong Polytechnic University, Hong Kong, China (People’s Republic); The Hong Kong Polytechnic University, Hong Kong, China (People’s Republic); The Hong Kong Polytechnic University, Hong Kong, China (People’s Republic)

## Abstract

Neurovascular coupling (NVC) dysfunction and gait disturbances are common in individuals with mild cognitive impairment (MCI), particularly under dual-task conditions requiring simultaneous cognitive and motor performance. This cross-sectional study compared gait parameters, NVC activation, and cognitive performance between 66 MCI participants and 47 healthy controls (mean age = 70.88 ± 4.66 years, 56% female) during three tasks: simple walking, the auditory clock test while walking, and the auditory Stroop test while walking. Gait parameters were recorded using APDM Mobility Lab sensors, cognitive performance (accuracy, reaction time, omission rate) was assessed via PsychoPy software, and NVC activation was measured using functional near-infrared spectroscopy. Feature selection and modeling were performed using Random Forest and Least Absolute Shrinkage and Selection Operator (LASSO) regression. In the simple walking task, MCI participants showed reduced turn counts and asymmetry in the terminal double support phase. During the auditory clock dual-task test, they exhibited reduced cognitive accuracy, incomplete turning, prolonged gait cycle duration, cadence asymmetry, terminal double support phase asymmetry, and increased activation in the left subcentral area. The auditory Stroop test revealed reduced cognitive accuracy, cadence, turn counts, elevated omission rates, stance and swing phase asymmetries, and increased activation in the left subcentral area, right dorsolateral prefrontal cortex, and right frontal pole area. These findings highlight the potential of combining gait parameters, NVC activation, and cognitive performance during auditory dual-task walking tests, particularly the auditory Stroop Test, as promising biomarkers for early detection of MCI. However, a larger sample is needed to enhance these findings’ robustness.

